# Shifting Public Interest in Liver Health, Hepatotoxic Medications, and Dietary Supplements in Kazakhstan: A Five-Year Google Trends Infodemiology Study

**DOI:** 10.3390/ijerph23081054

**Published:** 2026-08-14

**Authors:** Jamilya Saparbay, Zhandos Burkitbayev

**Affiliations:** 1 Department of Surgery, School of Medicine, Nazarbayev University, Astana 010000, Kazakhstan; 2 LLP “National Research Oncology Center”, Astana 010000, Kazakhstan; zhan11@mail.ru

**Keywords:** drug-induced liver injury, infodemiology, Google Trends, dietary supplements, Kazakhstan, pharmacovigilance, information-seeking behavior

## Abstract

**Highlights:**

**Public health relevance—How does this work relate to a public health issue?**

- This is the first infodemiology study on DILI in Central Asia, using Google Trends data from Kazakhstan (2021–2026).
- Dietary supplement searches showed a significant increasing trend while DILI symptom searches declined.

**Public health significance—Why is this work of significance to public health?**

- Rising public interest in unregulated dietary supplements represents a growing and under-recognized risk for hepatotoxicity in Kazakhstan.
- Google Trends is a viable low-cost tool for digital pharmacovigilance in low- and middle-income countries.

**Public health implications—What are the key implications or messages for practitioners, policy makers and/or researchers in public health?**

- Health authorities should strengthen regulation and hepatotoxicity labeling of dietary supplements.
- Digital surveillance should be integrated into clinical DILI monitoring systems in Central Asia.

**Abstract:**

**Background:** Drug-induced liver injury (DILI) is a leading cause of acute liver failure worldwide, yet epidemiological data from Central Asia remain scarce. Infodemiology—the study of online health information-seeking behavior—offers a novel approach to population-level health surveillance. **Objective:** Our aim was to investigate online information-seeking behavior related to DILI, hepatotoxic medications, and dietary supplements among the population of Kazakhstan using Google Trends data over a five-year period. **Methods:** A quantitative, retrospective, observational infodemiology study was conducted using Google Trends data from Kazakhstan (May 2021–May 2026). A total of 21 search terms were organized into five thematic sets: DILI symptoms, hepatotoxic medications, self-treatment and detoxification, liver-related symptoms, and general medication queries. Sets 1–4 were searched in Russian and Set 5 in Kazakh. Descriptive statistics, the Mann–Kendall trend test, and Spearman’s rank correlation analysis were performed using PAST software version 5. **Results:** A statistically significant trend was identified for 20 out of 21 search terms (*p* < 0.05). The majority of terms showed decreasing trends over the study period. In contrast, dietary supplements (BAD) demonstrated the strongest increasing trend (S = +1275, Z = +7.93, *p* < 0.001). Search interest for the general term “liver” was the highest of all 21 terms (mean RSV 84.30), while specific DILI symptoms such as dark urine (mean 1.28) and skin itching (mean 0.07) showed minimal search activity. Strong positive correlations were found between paracetamol and antibiotic searches (rs = 0.812, *p* < 0.001), consistent with self-medication patterns. Dietary supplements showed significant negative correlations with all detox-related terms (rs = −0.650, *p* < 0.001). **Conclusions:** This is the first infodemiology study on DILI in Central Asia. The significant increase in dietary supplement searches, combined with the low public awareness of specific DILI symptoms, highlights a critical public health concern. These findings underscore the need for enhanced regulation of dietary supplements, targeted public health education, and the integration of digital surveillance tools into pharmacovigilance systems in Kazakhstan.

## 1. Introduction

The new era has brought new rules for both patients and medical practitioners. Healthcare represents a particularly sensitive domain in terms of information access. With the widespread availability of the Internet, highly sensitive health-related information has become easily accessible to the general population. The use of the internet for health information management predates the formal terminology of the field: people were searching for health-related content online long before the term “infodemiology” was coined [1]. In 2002, Professor Gunther Eysenbach introduced the term “infodemiology”, originally defining it as the study of the distribution and determinants of information and misinformation in the digital environment, with the aim of informing public health and health policy. His original goal was to understand how health-related information and misinformation spread online, in order to guide healthcare professionals and policymakers in navigating an increasingly complex information landscape. Since then, the field has expanded substantially, with numerous applications in epidemiological surveillance, public health monitoring, and the study of health-related information-seeking behavior [2].

Google Trends has been increasingly applied in infodemiology research across a range of health topics. Its application to hepatology has been demonstrated in studies of viral hepatitis and liver disease search patterns in Europe [3].

Drug- and herbal and dietary supplement (HDS)-induced liver injury is one of the leading causes of acute liver failure (ALF) in the world. The severity of drug-induced liver injury (DILI) varies widely, ranging from slightly elevated liver enzymes to fulminant hepatic failure, requiring urgent liver transplantation [4]. DILI diagnosis remains a diagnosis of exclusion, as histological verification is rarely performed, which frequently leads to underestimation of the true incidence rate [5]. Several studies from the United States, Europe and South Korea have reported the incidence of DILI as approximately 10–19 cases per 100,000 population annually. DILI occurrence correlates significantly with age and gender, drug use increases with age due to developed comorbidities, and the observed predominance in women in recent years may be partly explained by the growing use of non-prescription drugs and dietary supplements [6].

DILI incidence also varies between inpatient and outpatient settings [7]. Not all drugs are associated with severe DILI leading to acute liver failure; however, the most widely implicated agent drug associated with ALF is acetaminophen (paracetamol), particularly in cases of intentional or unintentional overdose [8].

Epidemiological data regarding DILI from Central Asia are scarce. Economic development, rising income levels, and the emergence of a middle class in developing countries may serve as predictors of increased HDS use, as greater purchasing power facilities access to unregulated supplements. Accordingly, the indication for liver transplantation attributable to DILI- and HDS-related hepatotoxicity has increased significantly in recent years [9].

The most commonly implicated prescription drugs include antimicrobials, non-steroidal anti-inflammatory drugs and statins. Among herbal and dietary supplements, the leading causes of HDS-induced liver injury include anabolic steroids and weight loss supplements. In Kazakhstan, over-the-counter analgesics (paracetamol, ibuprofen) and self-prescribed antibiotics represent particularly prevalent hepatotoxic agents given the widespread culture of self-medication.

Given the limited regional data and the growing role of the internet as a primary source of health information, this study aimed to investigate online-information-seeking behavior related to DILI among the population of Kazakhstan using Google Trends data.

## 2. Methods

### 2.1. Study Design

This study employed a quantitative, retrospective, observational infodemiology design using Google Trends, which is a publicly available online tool that provides normalized data on the relative frequency of search queries submitted to the Google search engine over a defined time period and geographic region. This study was conducted in accordance with established infodemiology reporting guidelines (Mavragani et al., 2020) [10] and the Strengthening the Reporting of Observational studies in Epidemiology (STROBE) (File S1) guidelines [11]. Specifically, the infodemiology criteria applied included: use of a publicly available digital data source, clearly defined search terms with clinical relevance, specified geographic region and time period, and appropriate non-parametric statistical methods for time-series analysis.

### 2.2. Data Source and Extraction

Data were extracted from Google Trends (https://trends.google.com accessed on: 25 May 2026) in May 2026. The search was restricted to the Republic of Kazakhstan as the geographic region, the “Health” topic category, and the “Web Search” search type. The time frame of the collected data covered the five-year period from May 2021 to May 2026. Data were downloaded in monthly intervals, in comma-separated values (CSV) format, directly from the Google Trends platform and subsequently imported into Microsoft Excel for processing and analysis.

Google Trends provides data as relative search volume (RSV), expressed on a normalized scale of 0 to 100, where 100 represents the peak search interest for a given term within the specified time period and geographic region, and 0 indicates insufficient data or negligible search interest. It is important to note that RSV values represent relative rather than absolute search volumes and are therefore not directly comparable across search sets or time periods.

### 2.3. Search Term Selection

Search terms were selected based on clinical relevance to drug-induced liver injury (DILI), including characteristic symptoms of hepatotoxicity, commonly used hepatotoxic medications, self-treatment and detoxification-related queries, and general medication search behavior. A total of 21 search terms were organized to five thematic sets. Set 1 comprised five terms related to DILI symptoms. Within Set 1, both ‘itching’ (зуд) and ‘skin itching’ (кoжа зудит) were included as separate search items, as they represent distinct search behaviors in Russian: ‘зуд’ is a general medical term for pruritus, while ‘кoжа зудит’ is a colloquial phrase more commonly used by lay users to describe the same symptom. This distinction was intentional, as infodemiology studies have demonstrated that lay populations tend to use colloquial rather than clinical terminology when searching for health information. Sets 2 through 5 comprised four terms covering hepatotoxic medications, self-treatment and detoxification, liver-related symptoms, and general medications queries, respectively. Sets 1 through 4 were searched in Russian, reflecting the predominant language of internet use in Kazakhstan. Set 5 was searched in Kazakh to capture the information-seeking behavior of the Kazakh-speaking population. Set 5 terms were selected to represent the most frequently searched general medication-related queries in Kazakhstan based on Google Trends autocomplete suggestions and the infodemiology literature on medication search behavior. Paracetamol and antibiotics were included due to their well-documented association with DILI, while tablets/pills and medications were included as broad lay-language terms capturing general medication interest among Kazakh-speaking users. All search terms and their English translations are presented in Table 1.

### 2.4. Statistical Analysis

All statistical analyses were performed using PAST (Paleontological Statistics) software version 5, freely available at https://www.nhm.uio.no/english/resources/past/ (accessed on: 1 June 2026). Descriptive statistics including minimum, maximum, mean, median, and standard deviation (SD) were calculated for each search term across the full study period.

Monotonic temporal trends in RSV data were assessed using the Mann–Kendall trend test, a non-parametric method appropriate for time-series data that does not require normality of distribution. The Mann–Kendall statistic (S) and standardized Z-score were calculated for each search term, with *p* < 0.05 considered statistically significant. A positive S value indicates an increasing trend, while a negative S value indicates a decreasing trend over the study period.

The Spearman’s rank correlation coefficient (rs) was calculated to assess pairwise relationships between search terms within each thematic set. Spearman’s rank correlation was selected as a non-parametric method appropriate for the ordinal and non-normally distributed nature of RSV data. Correlation coefficients were interpreted as follows: rs < 0.20 negligible, 0.21–0.40 weak, 0.41–0.60 moderate, 0.61–0.80 strong and >0.80 very strong. Statistical significance was set at *p* < 0.05 for all analyses.

Although RSV values are not directly comparable across search sets due to independent normalization, descriptive comparisons of mean RSV values across sets are presented for exploratory purposes only and should be interpreted with caution.

Data visualization was performed using Microsoft Excel (Microsoft Corporation, Redmond, WA, USA). Figures were generated by the authors using original data files.

## 3. Results

Descriptive statistics for 21 search terms across five thematic categories are presented in Table 2. The overall mean relative search volume (RSV) varied substantially across search terms and categories.

Among symptom-related queries (Set 1), the highest mean RSV was observed for itching (mean 78.20, SD 9.60), followed by jaundice (mean 21.75, SD 3.39) and liver pain (mean 9.25, SD 1.68). Notably, dark urine (mean 1.28, SD 1.19) and skin itching (mean 0.07, SD 0.31) demonstrated minimal search activity throughout the study period, suggesting low public awareness of these specific DILI-associated symptoms in Kazakhstan.

Among medication-related queries (Set 2), ibuprofen demonstrated the highest mean RSV (mean 57.13, SD 14.64), followed by paracetamol (mean 45.48, SD 13.87) and antibiotics (mean 36.05, SD 7.12). Analgesic showed the lowest mean RSV in this category (mean 16.30, SD 2.37).

Within the self-treatment and detoxification category (Set 3), dietary supplements and detox demonstrated the highest mean RSV values (mean 69.29, SD 14.26 and mean 68.66, SD 13.42, respectively), indicating consistently high public interest in these terms throughout the study period. Liver detox showed the lowest mean RSV in this category (mean 1.59, SD 2.22).

Among liver-symptom-related queries (Set 4), the search term liver demonstrated the highest mean RSV of all 21 search terms (mean 84.30, SD 6.70), followed by nausea (mean 28.00, SD 6.10). Pain in right side (mean 1.90) and bitter taste in mouth (mean 4.90) showed relatively low search activity.

Among general medication queries (Set 5), pills demonstrated the highest mean RSV (mean 80.02, SD 7.12), followed by medication/drugs (mean 10.97, SD 1.18), with antibiotics and paracetamol showing lower values in this context (mean 5.85 and 7.56, respectively).

### 3.1. Temporal Trends (Figure 1, Figure 2, Figure 3, Figure 4 and Figure 5)

**Figure 1:**
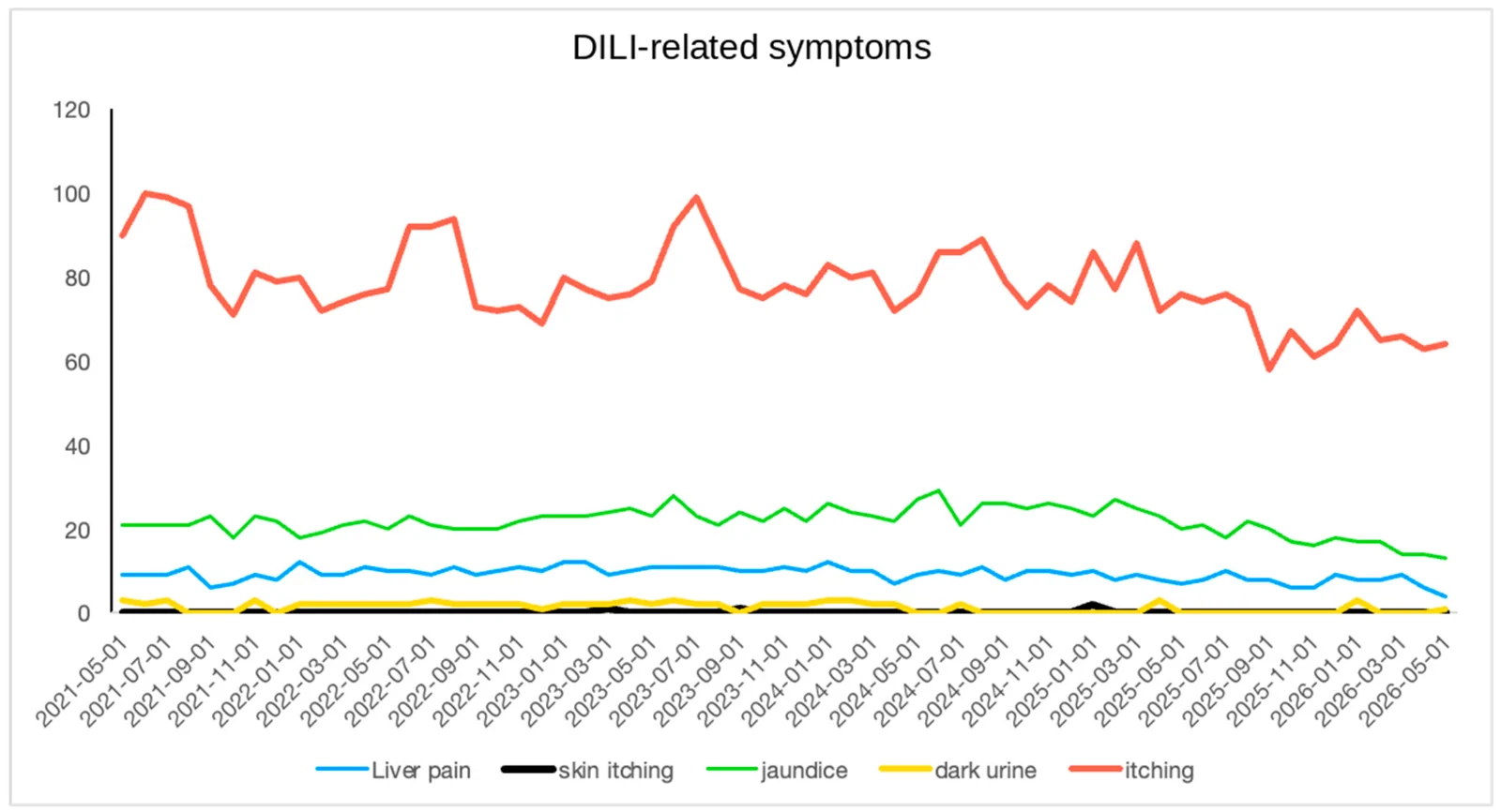
Temporal dynamics of DILI-symptom-related search terms. Monthly relative search volume (RSV) for DILI-related symptom queries on Google Trends in Kazakhstan, May 2021–May 2026. Search terms: itching (зуд), jaundice (желтуха), liver pain (печень бoлит), dark urine (темная мoча), skin itching (кoжа зудит). Scale 0–100.

Figure 1 illustrates the temporal dynamics of DILI-symptom-related search terms. The dominant search term was itching (зуд), which maintained consistently high RSV values throughout the period (range 58–100). Jaundice (желтуха) demonstrated moderate and relatively stable search interest (range 13–29), while liver pain, dark urine, and skin itching showed persistently low search volumes. Notably, the seasonal summer peaks in itching searches observed annually from 2021 to 2024 were absent in 2025, the reasons for which remain unclear. Possible explanations include changes in search behavior patterns, increased use of alternative information platforms such as Telegram or Instagram, or a genuine reduction in dermatological complaints. This observation warrants further investigation.

**Figure 2:**
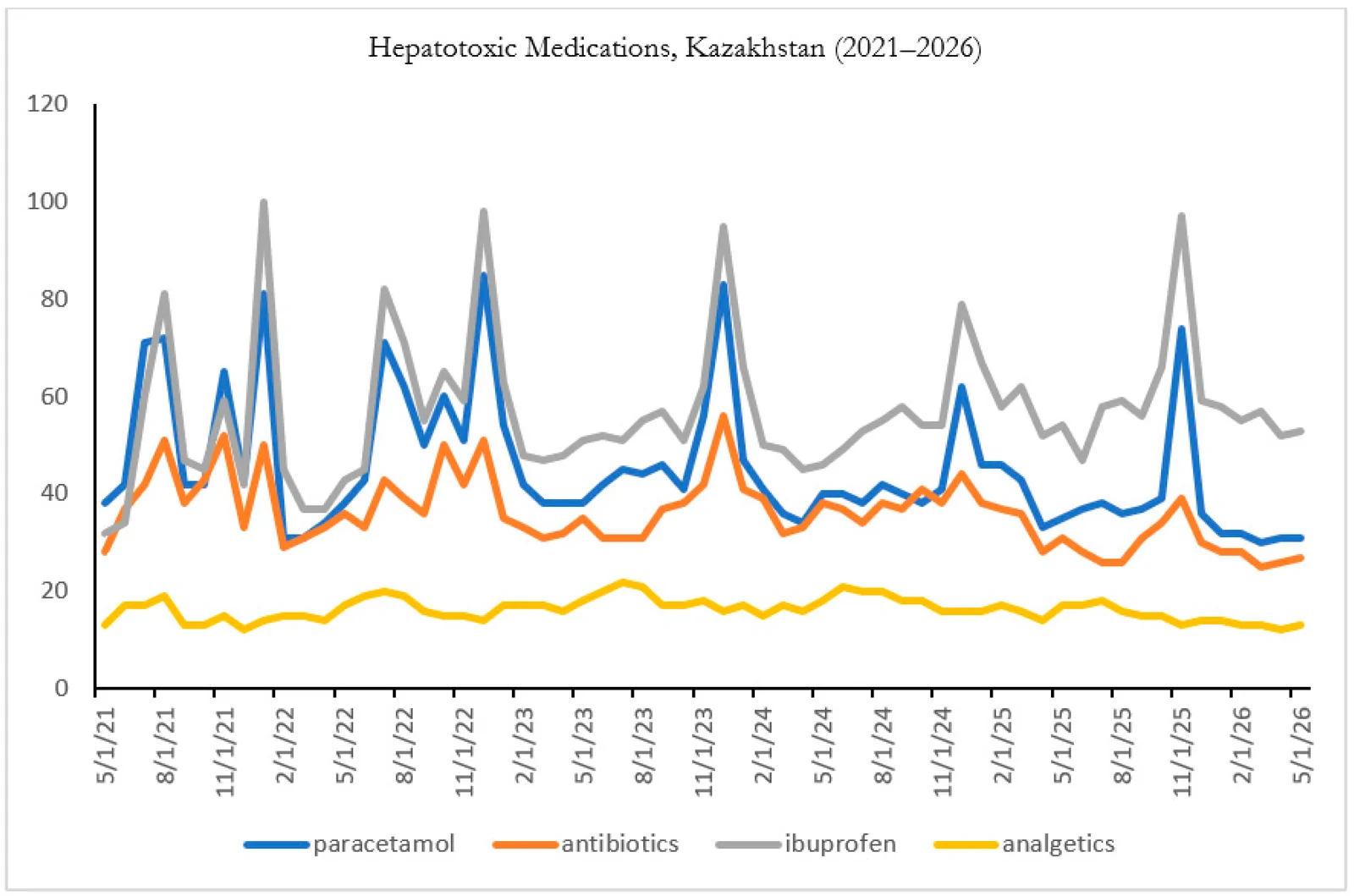
Search dynamics for hepatotoxic-medication-related terms. Monthly relative search volume (RSV) for potentially hepatotoxic over-the-counter medications (paracetamol, ibuprofen, analgesics) and antibiotics in Kazakhstan, retrieved from Google Trends™, May 2021–May 2026.

Figure 2 demonstrates search dynamics for hepatotoxic-medication-related terms. Ibuprofen showed the highest and most variable search volume (range 32–100), with pronounced peaks likely associated with acute respiratory infection seasons. Paracetamol and antibiotics demonstrated broadly parallel temporal patterns, consistent with their co-use during infectious illness episodes.

**Figure 3:**
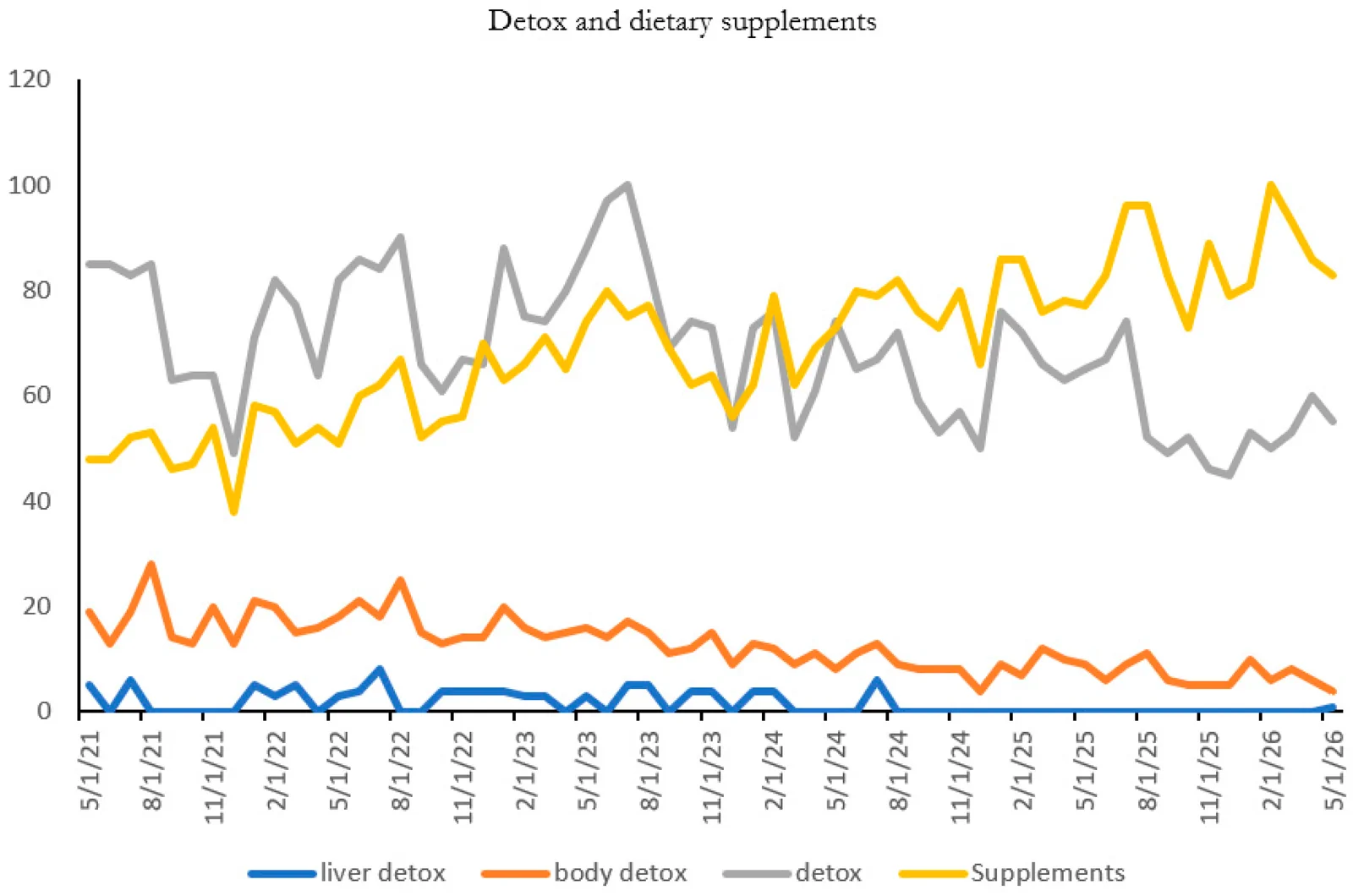
Search dynamics for self-treatment category. Monthly relative search volume (RSV) for detoxification-related queries and dietary supplements (BADs) in Kazakhstan, May 2021–May 2026. Search terms: liver cleansing (oчищение печени), body cleansing (oчищение oрганизма), detox (детoкс), dietary supplements (БАД). Scale 0–100.

Figure 3 reveals a striking divergence within the self-treatment category. While liver cleansing remained at near-zero levels throughout the study period, detox and dietary supplements (BADs) demonstrated high and sustained search interest. A visible upward trend in dietary supplement searches is apparent from 2023 onwards, contrasting the relatively stable or declining pattern for detox-related terms.

**Figure 4:**
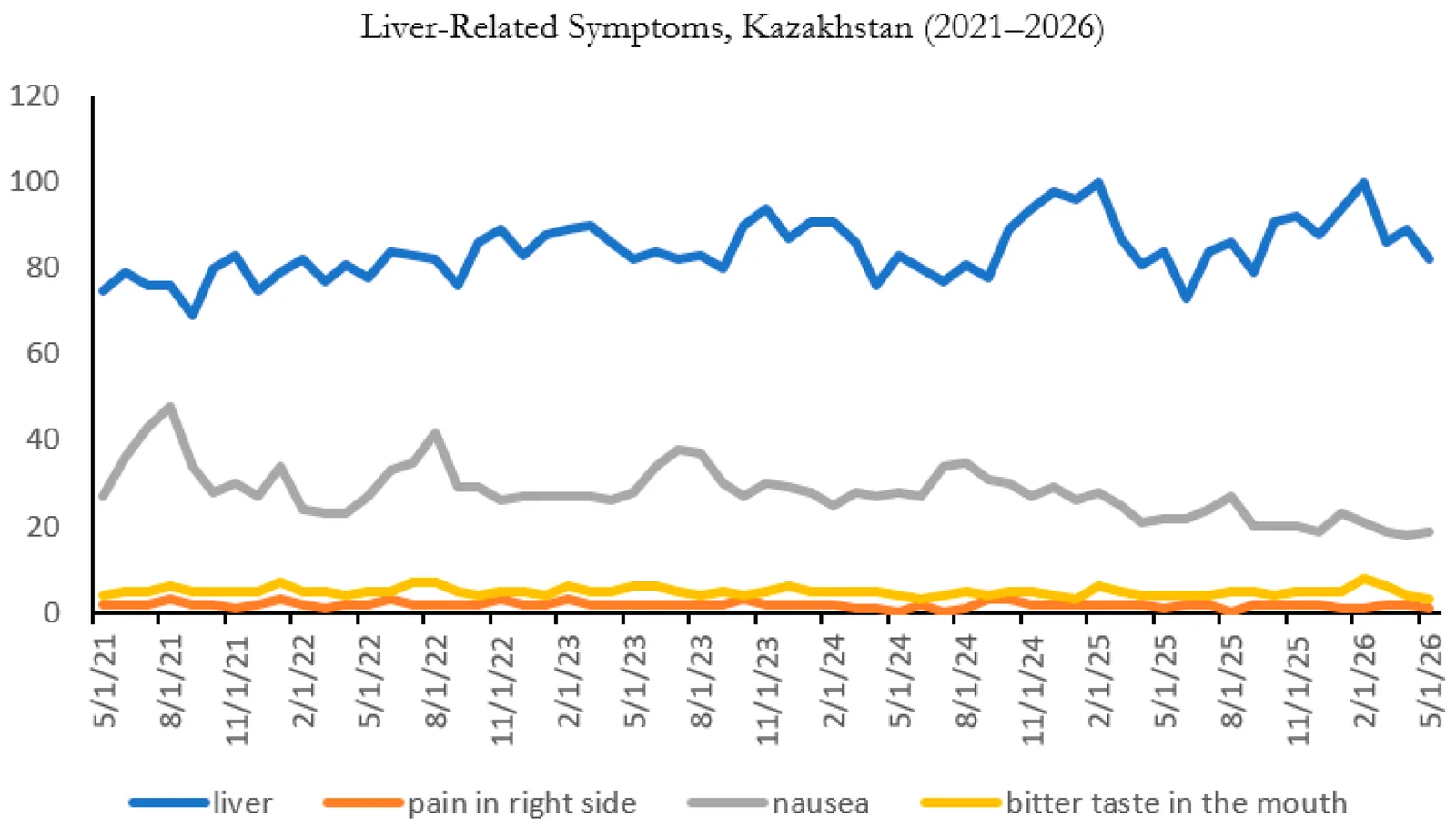
Search dynamics for liver-related symptoms. Monthly relative search volume (RSV) for liver-related symptom queries in Kazakhstan, May 2021–May 2026. Search terms: liver (печень), right hypochondrial pain (бoль в правoм пoдреберье), nausea (тoшнoта), bitter taste (гoречь вo рту). Scale 0–100.

Figure 4 shows that the general search term liver dominated this category, with consistently high RSV values (range 69–100). Nausea showed moderate search interest, with some variability, while the values for right hypochondrial pain and bitter taste in the mouth remained low throughout the study period.

**Figure 5:**
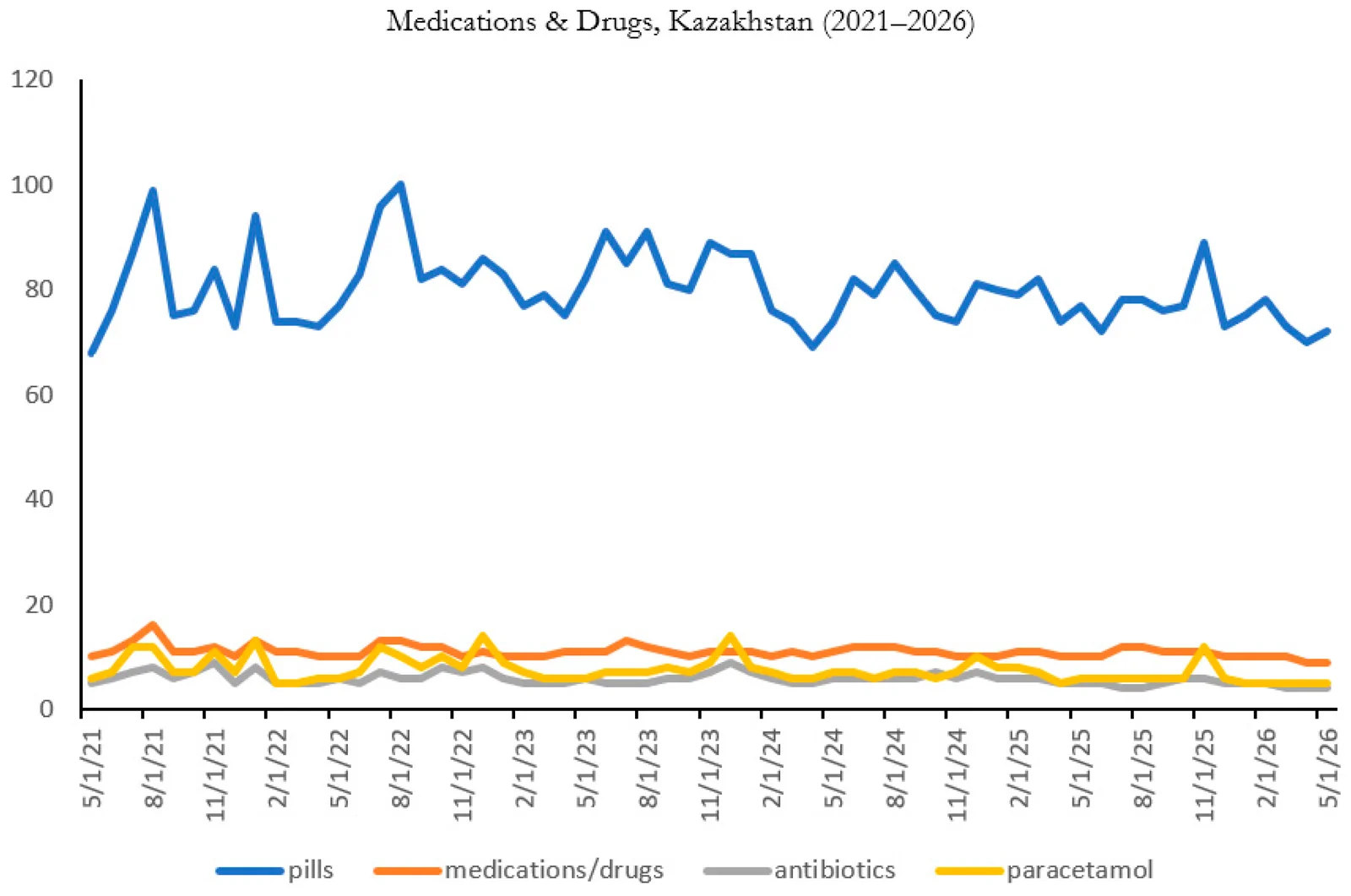
Search dynamics for general medication queries in Kazakh. Monthly relative search volume (RSV) for general medication-related search queries in Kazakhstan (Kazakh language), May 2021–May 2026. Search terms: tablets/pills (таблетки), medications (дәрілер), antibiotics (антибиoтиктер), paracetamol. Scale 0–100.

Figure 5 presents search dynamics for general medication queries in Kazakh. Pills demonstrated the highest search interest (range 68–100), with visible seasonal variation, while medications, antibiotics, and paracetamol showed relatively low and stable patterns throughout the period.

### 3.2. Trend Analysis

The Mann–Kendall trend test was performed for all 21 search terms across the five thematic categories. The results are presented in Table 3. A statistically significant trend was identified in 20 out of 21 search terms (*p* < 0.05).

Among symptom-related queries (set 1), all five search terms demonstrated statistically significant decreasing trends over the study period. The strongest decreasing trends were observed for itching (S = −644, Z = −4.01, *p* < 0.001) and dark urine (S = −644, Z = −4.01, *p* < 0.001), while liver pain, skin itching and jaundice showed moderate decreasing trends (s = −474, Z = −3.01, *p* = 0.003).

Among medication-related queries (Set 2), all four search terms—paracetamol, antibiotics, ibuprofen, and analgesics—demonstrated statistically significant decreasing trends (S = −591, Z = −3.68, *p* < 0.01 for all terms).

Within the self-treatment and detoxification category (Set 3), three out of four search terms showed statistically significant decreasing trends, including liver detox (S = −452, Z = −3.24, *p* = 0.001), body detox (S = −1181, Z = −7.36, *p* < 0.001), and detox (S = −715, Z = −4.45, *p* < 0.001). Notably, dietary supplements demonstrated a statistically significant increasing trend (S = +1275, Z = +7.93, *p* < 0.001), representing the only search term in this category with growing public interest over the study period.

Among liver-symptom-related queries (Set 4), liver and pain in the right side of the abdomen showed statistically significant increasing trends (S = +662, Z = +4.12, *p* < 0.001 for both terms), while nausea demonstrated a significant decreasing trend (S = −782, Z = −4.89, *p* < 0.001). Bitter taste in the mouth was the only search term across all five sets that showed no statistically significant trend (S = −290, Z = −1.96, *p* = 0.050).

Among general medication queries (Set 5), all four search terms demonstrated statistically significant decreasing trends, with paracetamol showing the strongest decrease (S = −540, Z = −3.44, *p* < 0.001), followed by antibiotics (S = −477, Z = −3.10, *p* = 0.002), medication/drugs (S = −399, Z = −2.61, *p* = 0.009), and pills (S = −378, Z = −2.35, *p* = 0.019).

### 3.3. Spearman’s Rank Correlation Analysis

Spearman’s rank correlation coefficients were calculated within each thematic set. Key findings are summarized below; full correlation matrices are presented in Supplementary Tables S1–S5.

Within Set 1 (DILI symptoms), significant positive correlations were observed between itching and liver pain (rs = 0.523, *p* < 0.001), itching and jaundice (rs = 0.449, *p* < 0.001), and itching and dark urine (rs = 0.329, *p* = 0.010), consistent with the clinical co-occurrence of cholestatic DILI symptoms.

Within Set 2 (medications), a strong positive correlation was found between paracetamol and antibiotics (rs = 0.812, *p* < 0.001), suggesting frequent co-searching consistent with self-medication patterns for infectious conditions.

Within Set 3 (self-treatment), dietary supplements (BADs) showed significant negative correlations with all detox-related terms, with the strongest inverse relationship observed with body detox (rs = −0.650, *p* < 0.001), reinforcing the Mann–Kendall finding of a diverging trend between supplement and detox searches.

Within Set 4 (liver symptoms), few significant correlations were identified, suggesting these symptoms were searched independently. Within Set 5 (general medication queries), all pairwise correlations were statistically significant (*p* < 0.05), with the strongest relationship between paracetamol and antibiotics (rs = 0.789, *p* < 0.001).

## 4. Discussion

This infodemiology study analyzed online-information-seeking behavior related to drug-induced liver injury in Kazakhstan over a five-year period (2021–2026) using Google Trends data. To the best of our knowledge, this is the first study to apply infodemiology methods to DILI surveillance in Central Asia. The findings reveal important patterns in public health awareness, self-medication behavior, and potential risk factors for hepatotoxicity in the Kazakhstan population. It is important to emphasize that Google Trends data reflect online search activity and should not be interpreted as a direct measure of public knowledge, health literacy, or actual seeking behavior. Search queries represent an intent to find information rather than confirmed awareness or understanding of a health topic. This distinction is particularly relevant when interpreting the observed trends in DILI symptom searches and dietary supplement interest, as increased search volume does not necessarily indicate informed decision-making or clinical presentation.

### 4.1. General Declining Trends in DILI-Related Searches

The most striking overall finding is the statistically significant decreasing trend observed in 20 out of 21 search terms. This pattern suggests a general decline in public information-seeking behavior related to liver disease symptoms and medications over the study period. Several explanations may account for this observation. First, the growing role of social media platforms such as Telegram and Instagram as a primary source of health information in Kazakhstan may have partially displaced traditional search engine use. Second, the post-COVID-19 period may have been associated with a normalization of health-seeking behavior. Third, improvements in healthcare accessibility may have reduced the need for online symptom searching.

In striking contrast to the general declining trend, searches for dietary supplements (BADs) demonstrated a statistically significant and robust increasing trend (S = +1275, Z = +7.93, *p* < 0.001)—the strongest trend observed in this study. This finding is of particular clinical significance in the context of DILI. Dietary supplements and herbal products represent a growing cause of drug-induced liver injury worldwide, and Kazakhstan’s regulatory framework for these products remains limited. The combination of increasing public interest in supplements and inadequate pharmacovigilance creates a potentially dangerous environment for hepatotoxicity.

Although our data did not allow the identification of specific supplement categories, market data provide important context. Vitamins C and D represent the most dynamic supplement categories in Kazakhstan, driven by post-COVID-19 immunity awareness, alongside probiotics, omega fatty acids, and combination herbal preparations. The Kazakhstani dietary supplement market reached USD 195.4 million in 2023, with over 90% of respondents reporting having taken supplements at least once, suggesting that the growing search interest reflects a broad-based shift in consumer health behavior rather than interest in any single product category [12,13].

The consistently high mean RSV values for supplements (mean 69.29) and detox products (mean 68.66) throughout the study period suggest that self-treatment with unregulated products is deeply embedded in health-seeking behavior in Kazakhstan. This phenomenon, combined with the strong negative correlation between supplement- and detox-related searches, indicates a temporal shift in preferences: as traditional detox searches decline, interest in commercial dietary supplements grows.

The statistically significant increasing trends for liver (S = +662, *p* < 0.001) and pain in the right side (S = +662, *p* < 0.001) suggest a growing public awareness of liver-related health concerns. The search term liver had the highest mean RSV of all 21 terms (mean 84.30), indicating persistent public interest in liver health. However, the relatively low search volumes for more specific DILI symptoms such as dark urine (mean 1.28) and jaundice (mean 21.75) suggest that while the public is aware of the liver as an organ of concern, knowledge of specific DILI symptoms remains limited. This gap between general liver awareness and specific symptom recognition represents an important target for public health education.

The high search volumes for ibuprofen (mean 57.13) and paracetamol (mean 45.48) indicate sustained public interest in these commonly used hepatotoxic medications. The strong correlation between paracetamol and antibiotic searches (rs = 0.812, *p* < 0.001) reflects a widespread pattern of self-medication for infectious conditions, which represents a significant risk factor for DILI. These findings underscore the importance of monitoring OTC medication use patterns and incorporating digital surveillance data into pharmacovigilance systems in Kazakhstan.

### 4.2. Limitations

Several limitations should be acknowledged. Google Trends™ data represent relative rather than absolute search volumes and cannot be converted into incidence estimates. The mixed-language search strategy—Sets 1–4 in Russian and Set 5 in Kazakh—may have introduced heterogeneity, as the RSV values in Set 5 reflect a partially distinct user group. Future studies should systematically compare Russian and Kazakh search patterns across all thematic categories to fully capture the bilingual nature of online health information-seeking in Kazakhstan. This study was limited to Google Trends data and did not include other search engines popular in Kazakhstan. Although alternative search engines such as Yandex are used in Kazakhstan, Google was selected as the sole data source to ensure comparability with international infodemiology studies, the majority of which rely exclusively on Google Trends data.

Seasonal variation was not formally adjusted for in the present analysis. As Kazakhstan experiences pronounced influenza seasons during winter months, the observed peaks in paracetamol, ibuprofen, and antibiotic searches likely reflect increased use of these medications during acute respiratory infection episodes rather than liver-specific health concerns. Future studies should incorporate seasonal decomposition methods to distinguish DILI-related search behavior from seasonally driven medication searches.

The selected symptom-related search terms are not specific to DILI and may reflect public interest in other liver disease sharing similar clinical manifestation, such as viral hepatitis or cholestasis.

The findings have several public health implications. The increasing trend in dietary supplement searches highlights the need for enhanced regulation, mandatory hepatotoxicity labeling, and targeted public awareness campaigns in Kazakhstan. Future research should incorporate Kazakh-language search terms, validate digital signals against clinical DILI registry data, and extend the analysis to social media platforms widely used in Kazakhstan such as Telegram and Instagram. The integration of patient survey data and real-world clinical DILI cases would further strengthen the correlation between digital signals and actual DILI burden.

## 5. Conclusions

This study demonstrates that Google Trends™ can serve as a valuable and accessible, and low-cost tool for monitoring DILI-related information-seeking behavior at the population level. Analyzing 21 search terms across five thematic categories in Kazakhstan over a five-year period (2021–2026), we identified a significant and sustained increase in dietary supplement searches against a background of declining interest in DILI symptoms and hepatotoxic medications. Concurrently, the growing public interest in general liver health, reflected by increasing search trends for ‘liver’ and ‘pain in right side’, contrasts the persistently low awareness of specific DILI symptoms such as dark urine and jaundice, highlighting a critical gap in public health literacy.

These findings have direct implications for public health policy in Kazakhstan and the broader Central Asian region. First, regulatory authorities should prioritize mandatory hepatotoxicity labeling and stricter oversight of the dietary supplements currently available without prescription. Second, public health campaigns should target awareness of specific DILI symptoms to enable earlier recognition and healthcare seeking. Third, digital surveillance tools, including Google Trends analysis, should be systematically integrated into national pharmacovigilance frameworks as complements to clinical reporting systems. Future research should validate these digital signals against clinical DILI registry data and extend the analysis to Kazakh-language search terms and social media platforms to achieve a more comprehensive picture of health-information-seeking behavior in Kazakhstan.

## Figures and Tables

**Table 1:** Search terms used in Google Trends analysis by thematic set, Kazakhstan, May 2021–May 2026.

Set	Сategory	Search Term (Russian)	Search Terms (English Translation)	Language
1	**DILI symptoms**	Зуд, желтуха, печень бoлит, темная мoча, кoжа зудит	Itching, jaundice, liver pain, dark urine, skin itching	Russian
2	**Hepatotoxic medications**	Парацетамoл, антибиoтики, ибупрoфен, анальгетики	Paracetamol, antibiotics, ibuprofen, analgesics	Russian
3	**Self-treatment and detoxification**	Очищение печени, oчищение oрганизма, детoкс, БАД *	Liver cleansing, body cleansing, detox, dietary supplements	Russian
4	**Liver-related symptoms**	Печень, бoль в правoм пoдреберье, тoшнoта, гoречь вo рту	Liver, right hypochondrial pain, nausea, bitter taste in mouth	Russian
5	**General medication queries**	Дәрілер, антибиoтиктер парацетамoл, таблетки	Tablets/pills, medications, antibiotics, paracetamol	Kazakh

Note: Sets 1–4 were searched in Russian; Set 5 was searched in Kazakh; * dietary supplements (БАД, in Russian).

**Table 2:** Descriptive statistics of relative search volume (RSV) for DILI-related search terms in Kazakhstan, May 2021–May 2026.

Search Item	Category	Min	Max	Mean	Median	SD
**Liver pain**	Symptoms	4.00	12.00	9.25	9.00	1.68
**Skin itching**	Symptoms	0.00	2.00	0.07	0.00	0.31
**Jaundice**	Symptoms	13.00	29.00	21.75	22.00	3.39
**Dark urine**	Symptoms	0.00	3.00	1.28	2.00	1.19
**Itching**	Symptoms	58.00	100.00	78.20	77.00	9.60
**Paracetamol**	Medications	30.00	85.00	45.48	41.00	13.87
**Antibiotics**	Medications	25.00	56.00	36.05	36.00	7.12
**Ibuprofen**	Medications	32.00	100.00	57.13	55.00	14.64
**Analgesics**	Medications	12.00	22.00	16.30	16.00	2.37
**Liver detox**	Self-treatment	0.00	8.00	1.59	0.00	2.22
**Body detox**	Self-treatment	4.00	28.00	12.54	13.00	5.27
**Detox**	Self-treatment	45.00	100.00	68.66	67.00	13.42
**Supplements**	Self-treatment	38.00	100.00	69.29	70.00	14.26
**Liver**	Symptoms	69.00	100.00	84.30	83.00	6.70
**Pain in right side**	Symptoms	0.00	3.00	1.90	2.00	0.70
**Nausea**	Symptoms	18.00	48.00	28.00	27.00	6.10
**Bitter taste in mouth**	Symptoms	3.00	8.00	4.90	5.00	1.00
**Pills**	General drugs	68.00	100.00	80.02	79.00	7.12
**Medication/drugs**	General drugs	9.00	16.00	10.97	11.00	1.18
**Antibiotics**	General drugs	4.00	9.00	5.85	6.00	1.17
**Paracetamol**	General drugs	5.00	14.00	7.56	7.00	2.33

Values represent normalized search volume on a scale of 1–100, where 100 indicates peak search interest within the specific time period. SD, standard deviation. Data retrieved from Google Trends.

**Table 3:** Trend analysis (Mann–Kendall test). Results of Mann–Kendall trend test for DILI-related search terms in Kazakhstan, May 2021–May 2026.

Search Item	Category	S	Z	*p*-Value	Trend
Liver pain	Symptoms	−474	−3.01	0.003	Decreasing
Skin itching	Symptoms	−474	−3.01	0.003	Decreasing
Jaundice	Symptoms	−474	−3.01	0.003	Decreasing
Dark urine	Symptoms	−644	−4.01	<0.001	Decreasing
Itching	Symptoms	−644	−4.01	<0.001	Decreasing
Paracetamol (Set 2)	Medications	−591	−3.68	<0.001	Decreasing
Antibiotics (Set 2)	Medications	−591	−3.68	<0.001	Decreasing
Ibuprofen	Medications	−591	−3.68	<0.001	Decreasing
Analgesics	Medications	−591	−3.68	<0.001	Decreasing
Liver detox	Self-treatment	−452	−3.24	0.001	Decreasing
Body detox	Self-treatment	−1181	−7.36	<0.001	Decreasing
Detox	Self-treatment	−715	−4.45	<0.001	Decreasing
Supplements	Self-treatment	+1275	+7.93	<0.001	Increasing
Liver	Symptoms	+662	+4.12	<0.001	Increasing
Pain in right side	Symptoms	+662	+4.12	<0.001	Increasing
Nausea	Symptoms	−782	−4.89	<0.001	Decreasing
Bitter taste in mouth	Symptoms	−290	−1.96	0.050	No statistically significant trend
Pills (Set 5)	General drugs	−378	−2.35	0.019	Decreasing
Medication/drugs (Set 5)	General drugs	−399	−2.61	0.009	Decreasing
Antibiotics (Set 5)	General drugs	−477	−3.10	0.002	Decreasing
Paracetamol (Set 5)	General drugs	−540	−3.44	<0.001	Decreasing

Note: S, Mann-Kendall statistic; Z, standardized test statistic; *p* < 0.05 considered statistically significant.

## Data Availability

The datasets generated and analyzed during the current study are publicly available from Google Trends (https://trends.google.com accessed on: 25 May 2026). Additional processed data are available from the corresponding author upon reasonable request.

## Supplementary Materials

The following supporting information can be downloaded at: https://www.mdpi.com/article/10.3390/ijerph23081054/s1, File S1: STROBE Checklist; Table S1: Spearman’s rank correlation matrix—Set 1 (DILI symptoms), Kazakhstan, May 2021–May 2026; Table S2: Spearman’s rank correlation matrix—Set 2 (Hepatotoxic medications), Kazakhstan, May 2021–May 2026; Table S3: Spearman’s rank correlation matrix—Set 3 (Self-treatment and detoxification), Kazakhstan, May 2021–May 2026; Table S4: Spearman’s rank correlation matrix—Set 4 (Liver-related symptoms), Kazakhstan, May 2021–May 2026; Table S5: Spearman’s rank correlation matrix—Set 5 (General medication queries), Kazakhstan, May 2021–May 2026.
