# Supplementary material for: Shifting Public Interest in Liver Health, Hepatotoxic Medications, and Dietary Supplements in Kazakhstan: A Five-Year Google Trends Infodemiology Study
Source: Int J Environ Res Public Health. 2026 Aug 14;23(8):1054. doi: 10.3390/ijerph23081054 (PMC13513398; doi:10.3390/ijerph23081054)
Supplement: Supplementary file 1 [file ijerph-23-01054-s001.zip › Supplementary_File_S1.pdf]

STROBE Statement — Checklist of Items for Reporting Observational Studies (Cross-Sectional / Infodemiology Design)

Manuscript: Rising Public Interest in Dietary Supplements Amid Declining DILI Symptom Searches in Kazakhstan: A Five-Year Google Trends™ Infodemiology Study

Item No.	Topic	STROBE Recommendation	Page	How addressed in this manuscript
**Title and Abstract**				
**1a**	**Title/abstract**	Indicate the study's design with a commonly used term in the title or abstract.	1	The abstract identifies the study as a "quantitative, retrospective, observational infodemiology study." The title references the Google Trends™ analysis design.
**1b**	**Title/abstract**	Provide in the abstract an informative and balanced summary of what was done and what was found.	1	The structured abstract (Background, Objective, Methods, Results, Conclusions) summarizes the design, data source, statistical methods, and key findings, including both the predominant decreasing trends and the increasing trend in dietary supplement searches.
**Introduction**				
**2**	**Background/rationale**	Explain the scientific background and rationale for the investigation being reported.	2	Section 1 (Introduction) describes the global burden of DILI, the role of infodemiology, and the scarcity of epidemiological data on DILI in Central Asia.
**3**	**Objectives**	State specific objectives, including any pre-specified hypotheses.	2	The final paragraph of the Introduction states the objective: to investigate online information-seeking behavior related to DILI among the population of Kazakhstan using Google Trends™ data over a five-year period.
**Methods**				
**4**	**Study design**	Present key elements of study design early in the paper.	3	Section 2.1 explicitly describes the study as a quantitative, retrospective, observational infodemiology design, conducted in accordance with Mavragani et al. (2020) infodemiology guidelines and STROBE.
**5**	**Setting**	Describe the setting, locations, and relevant dates, including periods of	3	Section 2.2 specifies the geographic setting

Item No.	Topic	STROBE Recommendation	Page	How addressed in this manuscript
		recruitment, exposure, follow-up, and data collection.		(Republic of Kazakhstan), data source (Google Trends™, "Health" category, "Web Search"), and the data collection period (May 2021–May 2026), with extraction performed in May 2026.
**6a**	**Participants**	Give the eligibility criteria, and the sources and methods of selection of participants.	3	Not applicable in the traditional sense — this study does not involve human participants. The unit of analysis is aggregated, anonymized search query data from Google Trends™ for the entire population of Kazakhstan, as detailed in Section 2.2 and 2.5 (Ethical considerations).
**7**	**Variables**	Clearly define all outcomes, exposures, predictors, potential confounders, and effect modifiers. Give diagnostic criteria, if applicable.	3–4	Section 2.3 and Table 1 define all 21 search terms (the outcome variable being relative search volume, RSV) organized into five thematic sets: DILI symptoms, hepatotoxic medications, self-treatment/detoxification, liver-related symptoms, and general medication queries. Search categories and language (Russian/Kazakh) are specified for each set.
**8**	**Data sources/measurement**	For each variable of interest, give sources of data and details of methods of assessment. Describe comparability of assessment methods if more than one group.	3	Section 2.2 describes Google Trends™ as the sole data source, the normalization method (RSV scale 0–100), and notes that RSV values are relative and not directly comparable across different search sets or time periods.
**9**	**Bias**	Describe any efforts to address potential sources of bias.	3, 9	Section 2.6 (Limitations, in Discussion) addresses potential sources of bias, including language bias (Russian-language predominance, partially mitigated by the inclusion of a Kazakh-language search set), national-level data aggregation, and changes in internet penetration over time.
**10**	**Study size**	Explain how the study size was arrived at.	3	The study size corresponds to the full available Google Trends™ time series for Kazakhstan over the maximum feasible

Item No.	Topic	STROBE Recommendation	Page	How addressed in this manuscript
				comparable period (60 monthly/weekly data points, May 2021–May 2026), as described in Section 2.2.
**11**	**Quantitative variables**	Explain how quantitative variables were handled in the analyses. If applicable, describe which groupings were chosen and why.	3–4	Section 2.3 explains the grouping of 21 search terms into 5 thematic sets based on clinical relevance to DILI. Section 2.4 describes RSV as a continuous variable (0–100) analyzed using descriptive statistics, without further categorization.
**12a**	**Statistical methods**	Describe all statistical methods, including those used to control for confounding.	4	Section 2.4 describes descriptive statistics (min, max, mean, median, SD), the Mann–Kendall trend test for monotonic trends, and Spearman's rank correlation for inter-term associations. As an ecological infodemiology study using aggregated search data, formal confounder adjustment was not applicable; this is acknowledged in the Limitations.
**12b**	**Statistical methods**	Describe any methods used to examine subgroups and interactions.	4	Subgroup analysis was performed by thematic search-term set (Sets 1–5) and by individual search term, as presented in Tables 2–3 and Section 3.4 (Spearman correlation by set).
**12c**	**Statistical methods**	Explain how missing data were addressed.	3	Not applicable — Google Trends™ provided complete time series data for all 21 search terms over the full study period with no missing values.
**12d**	**Statistical methods**	If applicable, describe analytical methods taking account of sampling strategy.	—	Not applicable. Google Trends™ data represent an algorithmically sampled and normalized aggregate of search queries; no separate sampling strategy was applied by the authors.
**12e**	**Statistical methods**	Describe any sensitivity analyses.	—	No additional sensitivity analyses were performed beyond the trend (Mann–Kendall) and correlation (Spearman) analyses described in Section 2.4. This is noted as an area

Item No.	Topic	STROBE Recommendation	Page	How addressed in this manuscript
				for future research in Section 4.6.
**Results**				
13a	**Participants**	Report numbers of individuals at each stage of study (e.g., potentially eligible, examined for eligibility, confirmed eligible, included in the study, completing follow-up, and analysed).	—	Not applicable — no individual human participants were recruited. All available Google Trends™ data points for the defined search terms and time period were included in the analysis (Section 2.2).
13b	**Participants**	Give reasons for non-participation at each stage.	—	Not applicable for the same reason as 13a.
13c	**Participants**	Consider use of a flow diagram.	—	Not applicable; a participant flow diagram is not relevant to this infodemiology study design.
14a	**Descriptive data**	Give characteristics of study participants (e.g., demographic, clinical, social) and information on exposures and potential confounders.	5–6	Table 2 (Section 3.1) provides descriptive statistics (min, max, mean, median, SD) for all 21 search terms across five thematic categories, serving as the descriptive characterization of the dataset.
14b	**Descriptive data**	Indicate number of participants with missing data for each variable of interest.	—	Not applicable — no missing data, as noted under item 12c.
14c	**Descriptive data**	Summarise follow-up time.	—	Not applicable in the traditional sense; the study period (60 months/weeks, May 2021–May 2026) is specified in Section 2.2 and serves as the temporal scope of analysis.
15	**Outcome data**	Report numbers of outcome events or summary measures.	6–9	Section 3.2 (temporal trends, Figures 1–5) and Section 3.3 (Table 3, Mann–Kendall results) report the trend statistics (S, Z, p-values) for all 21 search terms, including the direction (increasing/decreasing) and significance of each trend.
16a	**Main results**	Give unadjusted estimates and, if applicable, confounder-adjusted estimates and their precision (e.g., 95% CI). Make clear which confounders were adjusted for and why they were included.	6–9	Mann–Kendall S and Z statistics with exact or threshold p-values are reported for all 21 terms in Table 3 and Section 3.3. As an ecological infodemiology analysis, formal confounder

Item No.	Topic	STROBE Recommendation	Page	How addressed in this manuscript
				adjustment was not performed; this is addressed in Limitations (Section 4.5).
**16b**	**Main results**	Report category boundaries when continuous variables were categorized.	—	Not applicable — RSV was analyzed as a continuous variable (0–100 scale) and was not categorized.
**16c**	**Main results**	If relevant, consider translating estimates of relative risk into absolute risk for a meaningful time period.	—	Not applicable — Google Trends™ RSV data do not permit translation into absolute risk estimates, as explicitly discussed in Limitations (Section 4.5).
**17**	**Other analyses**	Report other analyses done — e.g., analyses of subgroups and interactions, and sensitivity analyses.	9–10	Section 3.4 reports Spearman's rank correlation analyses performed within each of the five thematic search-term sets, identifying significant positive and negative inter-term correlations (e.g., the negative correlation between dietary supplements and detox-related terms).
**Discussion**				
**18**	**Key results**	Summarise key results with reference to study objectives.	10	The opening paragraph of Section 4 (Discussion) summarizes the key findings — the predominant decreasing trend across most search terms, the significant increasing trend in dietary supplement searches, and increasing interest in liver-related symptoms — in direct reference to the study objective.
**19**	**Limitations**	Discuss limitations of the study, taking into account sources of potential bias or imprecision. Discuss both direction and magnitude of any potential bias.	12	Section 4.5 (Limitations) discusses the relative (non-absolute) nature of RSV data, language bias toward Russian-language searches, national-level data aggregation, the inability to account for changing internet penetration, and the impossibility of inferring causality.
**20**	**Interpretation**	Give a cautious overall interpretation of results considering objectives, limitations, multiplicity of analyses, results from similar studies, and other relevant evidence.	10–12	Sections 4.1–4.4 interpret each major finding (declining symptom searches, rising supplement interest, increasing liver-symptom searches, medication co-search

Item No.	Topic	STROBE Recommendation	Page	How addressed in this manuscript
				patterns) in the context of the study objectives, prior infodemiology literature (e.g., Gajdács et al.), and known DILI risk factors, while acknowledging the ecological nature of the data.
**21**	**Generalisability**	Discuss the generalisability (external validity) of the study results.	12–13	Section 4.6 (Future directions) and the Conclusion discuss the applicability of the findings to Kazakhstan and the broader Central Asian region, while noting that Russian-language predominance may limit generalisability to Kazakh-speaking subpopulations.
**Other Information**				
**22**	**Funding**	Give the source of funding and the role of the funders for the present study and, if applicable, for the original study on which the present article is based.	14	The Funding declaration states that this research did not receive any specific grant from funding agencies in the public, commercial, or not-for-profit sectors.

Note: This checklist is based on the STROBE Statement for cross-sectional studies, adapted for an infodemiology study design using publicly available, aggregated Google Trends™ data, in accordance with Mavragani et al. (2020). Page numbers refer to the manuscript section locations and may need adjustment to match final journal pagination.
