# Supplementary material for: Shifting Public Interest in Liver Health, Hepatotoxic Medications, and Dietary Supplements in Kazakhstan: A Five-Year Google Trends Infodemiology Study
Source: Int J Environ Res Public Health. 2026 Aug 14;23(8):1054. doi: 10.3390/ijerph23081054 (PMC13513398; doi:10.3390/ijerph23081054)
Supplement: Supplementary file 1 [file ijerph-23-01054-s001.zip › Supplementary_Tables_S1_S5.pdf]

Supplementary Materials

Shifting Public Interest in Liver Health, Hepatotoxic Medications, and Dietary Supplements in Kazakhstan: A Five-Year Google Trends Infodemiology Study

Jamilya Saparbay, Zhandos Burkitbayev

Table S1. Spearman's rank correlation matrix — Set 1 (DILI symptoms), Kazakhstan, May 2021–May 2026.

Search terms: itching (зуд), jaundice (желтуха), liver pain (печень болит), dark urine (темная моча), skin itching (кожа зудит). Russian-language searches.

	Liver pain	Skin itching	Jaundice	Dark urine	Itching
**Liver pain**	—	rs=0.054 p=0.681	**rs=0.385** p=0.002	**rs=0.356** p=0.005	**rs=0.523** p<0.001
**Skin itching**	rs=0.054 p=0.681	—	rs=0.177 p=0.173	rs=-0.122 p=0.348	rs=0.050 p=0.701
**Jaundice**	**rs=0.385** p=0.002	rs=0.177 p=0.173	—	rs=0.060 p=0.648	**rs=0.449** p<0.001
**Dark urine**	**rs=0.356** p=0.005	rs=-0.122 p=0.348	rs=0.060 p=0.648	—	**rs=0.329** p=0.010
**Itching**	**rs=0.523** p<0.001	rs=0.050 p=0.701	**rs=0.449** p<0.001	**rs=0.329** p=0.010	—

rs = Spearman's rank correlation coefficient. Green cells indicate statistically significant correlations (p<0.05). — = not applicable (diagonal). Data retrieved from Google Trends™, Kazakhstan, May 2021–May 2026.

Table S2. Spearman's rank correlation matrix — Set 2 (Hepatotoxic medications), Kazakhstan, May 2021–May 2026.

Search terms: paracetamol (парацетамол), antibiotics (антибиотики), ibuprofen (ибупрофен), analgesics (анальгетики). Russian-language searches.

	Paracetamol	Antibiotics	Ibuprofen	Analgesics
**Paracetamol**	—	**rs=0.812** p<0.001	**rs=0.591** p<0.001	rs=0.251 p=0.051
**Antibiotics**	**rs=0.812** p<0.001	—	**rs=0.461** p<0.001	rs=0.160 p=0.218
**Ibuprofen**	**rs=0.591** p<0.001	**rs=0.461** p<0.001	—	rs=0.038 p=0.771
**Analgesics**	rs=0.251 p=0.051	rs=0.160 p=0.218	rs=0.038 p=0.771	—

rs = Spearman's rank correlation coefficient. Green cells indicate statistically significant correlations (p<0.05). — = not applicable (diagonal). Data retrieved from Google Trends™, Kazakhstan, May 2021–May 2026.

Table S3. Spearman's rank correlation matrix — Set 3 (Self-treatment and detoxification), Kazakhstan, May 2021–May 2026.

Search terms: liver cleansing (очищение печени), body cleansing (очищение организма), detox (детокс), dietary supplements (БАД). Russian-language searches. Negative rs values indicate inverse correlations.

	Liver detox	Body detox	Detox	Supplements (BAD)
**Liver detox**	—	**rs=0.558** p<0.001	**rs=0.527** p<0.001	**rs=-0.314** p=0.014

	Liver detox	Body detox	Detox	Supplements (BAD)
**Body detox**	**rs=0.558** p<0.001	—	**rs=0.720** p<0.001	**rs=-0.650** p<0.001
**Detox**	**rs=0.527** p<0.001	**rs=0.720** p<0.001	—	**rs=-0.280** p=0.029
**Supplements (BAD)**	**rs=-0.314** p=0.014	**rs=-0.650** p<0.001	**rs=-0.280** p=0.029	—

rs = Spearman's rank correlation coefficient. Green cells indicate statistically significant correlations (p<0.05). — = not applicable (diagonal). Data retrieved from Google Trends™, Kazakhstan, May 2021–May 2026.

Table S4. Spearman's rank correlation matrix — Set 4 (Liver-related symptoms), Kazakhstan, May 2021–May 2026.

Search terms: liver (печень), right hypochondrial pain (боль в правом подреберье), nausea (тошнота), bitter taste in the mouth (горечь во рту). Russian-language searches.

	Liver	Pain in right side	Nausea	Bitter taste
**Liver**	—	rs=0.046 p=0.725	**rs=-0.296** p=0.021	rs=0.055 p=0.674
**Pain in right side**	rs=0.046 p=0.725	—	rs=0.191 p=0.140	rs=0.145 p=0.265
**Nausea**	**rs=-0.296** p=0.021	rs=0.191 p=0.140	—	**rs=0.289** p=0.024
**Bitter taste**	rs=0.055 p=0.674	rs=0.145 p=0.265	**rs=0.289** p=0.024	—

rs = Spearman's rank correlation coefficient. Green cells indicate statistically significant correlations (p<0.05). — = not applicable (diagonal). Data retrieved from Google Trends™, Kazakhstan, May 2021–May 2026.

Table S5. Spearman's rank correlation matrix — Set 5 (General medication queries), Kazakhstan, May 2021–May 2026.

Search terms: tablets/pills (таблетки), medications (дәрілер), antibiotics (антибиотиктер), paracetamol. Kazakh-language searches.

	Pills	Medications/drugs	Antibiotics	Paracetamol
**Pills**	—	**rs=0.634** p<0.001	**rs=0.601** p<0.001	**rs=0.767** p<0.001
**Medications/drugs**	**rs=0.634** p<0.001	—	**rs=0.422** p=0.001	**rs=0.459** p<0.001
**Antibiotics**	**rs=0.601** p<0.001	**rs=0.422** p=0.001	—	**rs=0.789** p<0.001
**Paracetamol**	**rs=0.767** p<0.001	**rs=0.459** p<0.001	**rs=0.789** p<0.001	—

rs = Spearman's rank correlation coefficient. Green cells indicate statistically significant correlations (p<0.05). — = not applicable (diagonal). Data retrieved from Google Trends™, Kazakhstan, May 2021–May 2026.
